# Researches in Comparative Pathology

**Published:** 1847-07

**Authors:** 


### Art. V.

*Recherches de Pathologie Comparée.* Par Ch. F. Heusinger.—*Cassel,* 1845.

*Researches in Comparative Pathology.* By C. F. Heusinger.—*Cassel,* 1845. 4to, pp. 155, DXLIX.

It affords us much pleasure to observe the advance that is being made in the study of comparative pathology. There can be but one opinion as to the importance of the subject, and the advantage to be derived by human pathology from a careful study of the diseases of animals; and when we see such men as Andral and Rayer turning their attention to it in good earnest, we have reason to expect that medical science will profit by their labours. The important researches of M. Andral on the blood of animals are already known to our readers. M. Rayer is at present publishing a Journal of Comparative Pathology: we notice occasionally, especially in the foreign medical journals, excellent articles on the same subject; and now it is our pleasing duty to introduce to the notice of our readers M. Heusinger's very valuable contribution to comparative pathology. This is, indeed, a remarkable production, the fruit of long and laborious literary research and of considerable practical observation and experience in the diseases of the lower animals. The title of the work but faintly indicates the nature and value of the contents; for it is not simply a treatise on those maladies which are common to man and the domestic animals, but contains in addition, an elaborate account of veterinary medicine from the earliest period down to the present; a history of the great epidemics of Europe, with valuable remarks; and a memoir on the diseases of birds: showing, as the author justly observes in his preface, that the monograph contains matter of interest not only to the physician and veterinarian, but also to the historian and student of natural history.

The physical life of man, says M. Heusinger, having the same conditions and exhibiting the same general phenomena as that of animals, must also present the same resemblance in the state of disease. We may, therefore, very naturally ask, what influence has the medicine of animals upon that of man; and what influence ought it to have? Such is the text with which the author sets out, and all his subsequent observations have for their object the elucidation of the interesting questions involved in it. Although it is the special object of one division of the work, ("Sketch of comparative nosography of man and of domestic animals,") to prove that very nearly the same diseases occur in veterinary as in human medicine; and that, therefore, the study of the one is calculated to throw much light upon that of the other; the author keeps the same object steadily in view in those parts of his memoir not so immediately connected with human medicine, and especially in his historical details; thus imparting a character of unity and closeness to the work which renders the subject easy to the student.

M. Heusinger proposes to discuss his subjects in the following order:

1. By comparing the development and progress of human and of veterinary medicine.

2. By comparing the diseases of man and those to which animals are subject.

3. By comparing the conditions and phenomena which accompany the development of disease in man and animals.

It would be impossible to embrace within the limits of an article suited to the space of this journal, an analysis of the different subjects here enumerated; we shall therefore, for the present, confine ourselves to one, namely, the "comparative nosography of man and animals," which we select as being the most immediately connected with human pathology.

The author requests the readers to bear in mind, while perusing this section of his work, that as yet we have no comparative nosography, and that we cannot form a complete one while the diseases of animals are so imperfectly understood and so ill-defined, even by veterinary writers. His object in drawing up the following "sketch" is to encourage others to pursue the inquiry further, and to throw out a few hints, as he moderately calls his valuable researches, which may be of use to his successors in their inquiries into the same interesting and unexplored field of science.

DIGESTIVE APPARATUS. *Diseases of the mouth.* The domestic animals are subject to several varieties of aphtha, more especially to an epizootic form, called the aphthongular disease. This malady is very common amongst the ruminants, but rarely attacks dogs, horses, and swine; it is contagious, and sometimes attacks the human species. A disease similar to stomatitis gangrenosa in man frequently attacks the lower animals, especially hogs. The tongue is the part principally involved, and when the disease occurs in an epizootic form, as it often does, it is very fatal. It is called by the Italian writers, "*Mul pinzanese, Cancro volante,*" &c. A disease to which birds are very subject, called anthrax of the gullet and windpipe, closely resembles angina gangrenosa in man. All the varieties of angina are frequently met with in the domestic animals. Cancer of the mouth, lips, or tongue is a very rare disease in animals, the horse alone being subject to it, and even he but seldom.

*Diseases of the stomach. Pneumatosis* or *tympanitis* is a common and very dangerous complaint amongst oxen, sheep, and goats. It is produced by eating freely of young grass and clover, the result of which is the formation of an immense quantity of gas in the first stomach, containing besides carbonic acid so much hydrogen and carburetted hydrogen, that it ignites instantly when brought in contact with a lighted taper.*

Vomiting, which may be regarded as a physiological act in the dog and in swine, is an indication of some serious disease of the stomach in ruminants, and in the horse it is almost always a mortal sign. Hæmatemesis is also observed in the same classes of animals, but less frequently in the two latter than the former. Ructitation is a common affection in horses, and, as in man, is generally a symptom of indigestion, or of organic disease of the stomach or intestinal canal. *Gastritis* is a very common disease amongst all domestic animals. Dogs are very subject to *gastritis mucosa* and often die of it. Horses, and especially ruminants, are frequently attacked by a dangerous form of *gastro-enteritis mucosa,* which is supposed to be produced by the use of wild or forest pasturage. The Germans call it wood-ill—"*waldkrankheit.*"

Scirrhus and cancer of the stomach are often met with in the horse, and sometimes in the ox. Perforation of the stomach by ulceration and gangrene occurs in domestic animals as well as in man; but rupture of the stomach, by mechanical causes, and without preceding disease, is a lesion almost confined to the horse and the ass. It is, however, sometimes the sequel of tympanitis in the ruminants. Calculi of considerable size have been found in the stomach of the dog, and even of the horse: their structure is not described. Intestinal worms are often found in the stomach of the lower animals, and sheep in particular suffer much from this accident.

*Diseases of the small intestines.* The different forms of enteritis are commonly met with in all the domestic animals, and they are very often complicated with gastritis. These animals are more subject to worms than man. In the small intestines of the horse they are not unfrequently found in immense numbers.

*Diseases of the large intestines.* The young of animals are subject to diarrhœa during the process of dentition precisely as children are. Dysentery is very common amongst domestic animals, both in an enzootic and epizootic form. In warm countries it is very fatal to animals as well as to man. Torsion of the intestines is very common and very fatal in some of the lower animals; it generally occurs in the cæcum and colon. Rupture of the large intestines, produced by constipation or other obstruc-

* We have seen several cases of this disease in the cow, in which it was supposed to be produced by the immoderate use of raw potatoes and turnips. The beast was immensely swollen, and, on the side being punctured, a stream of gas issued from the aperture, which readily ignited, and burned with a blue flame for a considerable time.—REV.

tion of the bowels, is by no means an unfrequent occurrence in the horse and ox. Prolapsus and fistula ani are almost as commonly met with in the dog and horse as in man. The large as well as the small intestines are very subject to worms, and they exist in enormous quantities.

LESIONS OF THE LYMPHATIC SYSTEM. M. Heusinger's observations lead him to the conclusion that young animals are as liable to disease of the lymphatic system as those of the human species,—especially the horse, in which this morbid condition easily degenerates into a general cachectic state of the system, in the event of which a simple cold or superficial ulceration will quickly merge into glanders or farcy. M. Heusinger justly remarks, that many obscure points in human pathology might be materially cleared up by the study and comparison of these morbid states in the two races.

*Diseases of the liver and bile.* Dogs are often attacked by chronic hepatitis, and sheep and oxen by an acute form of the disease, with ramollissement of the liver, which is a dangerous if not fatal malady. The same parasites which are found in the human liver are also commonly met with in the hepatic ducts of animals. The echinococcus and cysticercus are frequently found in the liver of ruminants and in swine. The disease called cirrhosis has not been observed, although M. Heusinger has no doubt of its existence.

Inflammation of the biliary ducts—a common disease amongst the ruminants—frequently terminates in a remarkable kind of ossification of these vessels. Jaundice is a common disease amongst dogs and sheep; the horse is rarely attacked. Biliary calculi are not so frequently found in animals as in man; in oxen and swine they are sometimes met with.

VASCULAR SYSTEM. Traumatic phlebitis, and that variety which is the result of the absorption of pus, are often discovered. Rupture of the veins, especially those near the surface, and ossification, also occur in the domestic animals; the arteries are seldom attacked by inflammation. Ossification is a rare occurrence; and aneurism is confined to the large trunks, particularly to those in the vicinity of the heart. Diseases of the heart are by no means uncommon in animals. Pericarditis is often met with: and there is a peculiar form of this disease, caused by pins or nails which the animal has swallowed, passing from the stomach through the diaphragm into the heart; this is generally mortal. Hydrops pericardii is a common affection amongst sheep and dogs. Endocarditis occurs in animals in connexion with articular rheumatism. Softening of the heart, and that condition commonly observed after typhoid fever in man are often found in our domestic animals. Hydatids, and even worms, cysticercus and strongylus, have been found in the heart of animals.

RESPIRATORY ORGANS. In all acute diseases of dogs the respiratory organs are sure to be attacked. Pleuritis, hydrops pleuræ, and pseudomorphosis, are very common, particularly in dogs and sheep. Acute and subacute pneumonia and pleuropneumonia, emphysema, and œdema pulmonum, are constant diseases. Tubercles of the lungs are common to all domestic animals, (but the author does not say whether the structure and composition of these degenerations are similar to those observed in phthisis pulmonalis in man). Gangrene of the lungs is much more common in animals than in man. Hydatids and parasites are constantly found in the lungs and air-passages of the domestic animals.

The catarrhal affections of animals present a striking analogy to those diseases in the human subject. They assume, however, in each species of animals certain peculiar and characteristic features. In the horse, the lymphatic ganglions are almost immediately inflamed and subsequently engorged, in which state the disease rapidly passes into glanders. In oxen, common catarrh is very liable to terminate in an ulcerated and gangrenous state of the parts, which is exceedingly dangerous. In sheep, this complaint often assumes a cachectic and even a putrid form, which the Germans call glanders of sheep. When catarrh attacks dogs, it has great tendency to become complicated with a peculiar nervous and atoxic state, which resembles the disease in the sheep. Cats are often similarly attacked.

The catarrhal affections in animals have a great tendency to become contagious, to pass from one species of animals to another, and to assume an epizootic form. For example, horses are frequently attacked by a disease very similar to the influenza in man, when that disease exists in an epidemic form amongst the human race.

THE SKIN. The great distinction which exists between the structure of the skin of animals and that of man, must necessarily cause some difference in the diseases of this tissue. The great mobility of the skin in the lower animals, the development of the cutaneous muscle, the existence of large quantities of cellular tissue, and of an exaggerated lymphatic system, under the skin, will explain the cause of these variations.

To understand the cutaneous diseases of animals, the veterinarian must be familiar with these affections in the human subject, and he who describes correctly the development and metamorphoses of a single disease, has done more for this branch of pathology than if he had invented a dozen new classifications.

Eczema is a common disease of sheep, although not described by authors. Under the name of herpes, many diseases have been described which have no connexion with it. True herpes, however, does occur in the horse and dog, and complicated with eczema in the sheep. The *bullæ* frequently attack animals, although not mentioned by writers, and one variety (a kind of rupia), is very troublesome to the horse, about the fetlock. Impetiginous eruptions frequently occur on the face of young animals; but they are so imperfectly described by veterinary writers that it is impossible to give their exact names. For example, it is difficult to tell whether the tinea contagiosa of cats is a porrigo or an impetigo. The porrigos are very common affections. A form of crusta lactea, similar to that observed in man, attacks calves and lambs, and is included amongst these eruptions. Porrigo of the feet is similar, if not identical, to the "grease" of veterinary writers, and the "mauke" and "eaux aux jambes" of the German and French authors; but so many diseases have been described and confounded under this term, that it is not easily individualized. It corresponds to the impetigo sparsa of man more than to any other disease. Favus has not been sufficiently clearly observed in animals. MM. Haubener and Greve, however, describe under the name of "porrigo decalvans equorum" an eruption similar to that which occurs in man; but the description is by no means clear. Variola is a common disease of animals. It does not, says M. Heusinger, originate with animals, but is transmitted to them from man; it passes readily from one animal, to another and from animals to man, and *vice versâ,* and the more severe the disease the greater is the guarantee against a second attack. When well developed it has the strongest resemblance to the variola of the human subject; but, as in man, it often occurs in a transitory form, with slight febrile symptoms, and without eruption; also in the form of variola varicella; and there is even a species of varioloid which attacks sheep. It is highly contagious, and has been observed in new-born lambs which had contracted it in the womb. Sheep, dogs, rabbits, and goats are subject to variola. The variola of hogs is well known; it occurs in an epizootic form when the disease prevails as an epidemic in the human race. M. Heusinger entertains the opinion that the vaccine disease (*vaccinia*) was first produced by the transmission of variola, either immediately from man or medially through animals to the cow, and that it did not originate in an epizootic form, and without external contact, as alleged by many writers.

Malignant pustule, or anthrax epizooticus, is one of the most common diseases. It attacks quadrupeds, fowls, fish, all the domestic animals, and is sporadic and highly contagious. This disease originates in animals of the equine species, and is transmissible to man. Its elementary nature is unknown, but is supposed to be decomposition or a vitiated state of the blood *sui generis.*

The squamous eruptions are often confounded in animals as in man, with the desquamation which occurs in eczema and other of the exanthematous affections. A mild form of pityriasis exists in animals in the healthy state; but it also occurs in an exaggerated form on a red base, constituting a disease of very frequent occurrence amongst horses and swine. Psoriasis occurs in several forms. In the horse, P. ophthalmica, P. podicis, P. pudendi, have been often observed; but more frequently, psoriasis labialis (the *crusta labialis* of some writers) and psoriasis of the joints and extremities. Lepra has not been described, although several diseases have been mistaken for it.

URINARY ORGANS. Nephritis, although not generally known, occurs in the horse; and M. Heusinger is of opinion that the albuminous disease also attacks that animal, although hitherto it has passed unnoticed.* Inflammation of the mucous lining of the bladder, and vesical catarrh are common affections of animals; the former occurs frequently in an epizootic form amongst dogs. Rupture of the bladder occurs sometimes in the horse. Hydruria is a frequent disease of the horse, and occurs not unfrequently amongst the ruminants. The majority of cases of diabetes of the horse are cases of hydruria; but our knowledge of the chemical analysis of the secretion is too imperfect to enable us to say whether or not it corresponds to diabetes melitus in man. M. Lassaigne states, in an account of an epizootic of this malady, which occurred at Paris, that he detected in the secretion a large quantity of acetic acid partly free, which is rather remarkable. This epizootic attacked almost exclusively entire horses, seldom those that were castrated, and not a single instance is recorded in which mares were attacked. In man, diabetes melitus rarely attacks the female sex, and seldom before the age of puberty. In hydruria of the horse, exactly the reverse occurs. Hydruria in man

* Messrs. Clayworth and Percivall have described "albuminuria" as a disease of the horse, in the 'Veterinarian' for 1841.—REV.

occurs as often in the female as in the male. This is a subject of considerable interest as regards human pathology. Hematuria sometimes attacks dogs, more frequently horses and swine; but sheep and cows, amongst which it occurs in an enzootic and epizootic form, are still more frequently attacked. Lithiasis of the kidneys and bladder are common diseases amongst our domestic animals. M. Heusinger has collected many cases of this malady, which he himself had observed; but his analysis of them is not sufficiently complete to enable him to arrive at general conclusions. He has observed copious sediment so frequently in the bladder of several of the ruminants, that he regards it as a natural condition in those animals. Ischuria, dysuria, and eneuresis are often met with in the horse and dog, and the former with the same characters as those it presents in man.

SEXUAL ORGANS. Sarcocele and scirrhus of the testicle have been frequently observed in dogs, horses, asses, and swine. Varicocele occurs only in the horse, and hydrocele rarely attacks any of the domestic animals. The prostate is sometimes hypertrophied and scirrhous in the dog. Blennorrhea, and cancer, and scirrhus of the penis have been observed in horses and dogs; and phimosis and paraphimosis in the horse, the dog, and the bull. Inflammation of the uterus occurs more commonly in mares and cows, than in women, and is accompanied by a mucous, muco-purulent, or purulent discharge, forming a kind of dropsy of the womb. Puerperal inflammation (*metritis puerperalis*) is also more common in the domestic animals than in women. Polypus of the uterus is often met with in mares and cows, scirrhus and cancer rarely; they have been observed, however, in cows and bitches. Monkeys, which sometimes show a discharge similar to menstruation in women, are subject to attacks of uterine hemorrhage. Uterine hemorrhage of a different character occurs in dogs, cows, and sheep. Prolapsus of the uterus is common amongst all domestic animals. Leucorrhœa is a frequent disease of cows, mares, and dogs. Scirrhus and carcinoma are as common in animals as in women. They occur perhaps more frequently in bitches than in any other animals.

OSSEOUS SYSTEM AND JOINTS. Periostitis and ostitis are very frequent diseases. Osteophyta have been observed in most of the domestic animals. They are very common amongst poultry; they are very frequently met with on the dorsal vertebra of oxen and horses, and also on the feet of the latter. Hydrops bursæ is common to all the domestic animals, and it occurs more frequently in the horse than in man. The small free cartilaginous bodies (*chondroides*) which are sometimes but rarely found in these tumours in man, are very frequently met with in the same tumours in the horse, and especially in the chamois. Arthritis is a very common affection of the horse, and is not unfrequent amongst the other domestic animals. Anchylosis has been observed in almost all the articulations of the domestic animals. In the horse especially, it is much more common than in man.

CACHECTIC DISEASES. The cachectic diseases of animals are in several respects very different from those of man; but still there is considerable resemblance between many of the diseases individually, and no doubt a more profound study of their nature and character in both classes of animals would disclose a much stronger analogy than that which we are now enabled to point out. Dropsical diseases (*cachexiæ aquosæ*) are common to all domestic animals, and are frequently met with in savage nature. They are often observed, at the same time and in the same place, in the endemic and enzootic, epidemic and epizootic forms. Some animals, however, are much more commonly attacked than others.

Scrofulous diseases occur in all animals, not only in quadrupeds but in birds, and under the same conditions and influences as in the human subject. They are generally enzootic, frequently hereditary, and occur in herbivorous animals more commonly than in the carnivori. They assume the same forms in animals as in man. Simple scrofula is a more common disease of animals than in the human subject, although this fact has escaped the observation of physicians. A very interesting and instructive article on the transition of the various forms of the scrofulous disease has been published by M. Erdt (Gurlt und Hertwig Magazin, vi, p. 292). A flock of 350 lambs were kept in a close and confined stable, badly ventilated, and were fed on very rich and succulent food; presently evident signs of scrofula were manifested, and as soon as these were observed the lambs were suddenly removed to a colder habitation, and put upon a less nutritious diet; the external symptoms vanished, but were immediately succeeded by a copious salivary discharge, atrophy, and rachitis, under which the animals died.

Each class of animals has a predisposition for certain forms of the disease; thus, in the horse the pituitary membrane is the part attacked; in the sheep the disease manifests itself in the form of rickets; in the calf it is confined to the skin, and in swine it almost invariably attacks the glandular system. It is contagious in animals. (?) Catarrhal affections have a great tendency to degenerate into scrofula of the glandular system, especially in fowls. Rachitis is a very common disease in quadrupeds, and even in birds. It is, in animals as in man, a disease of fœtal life or of the early periods of existence. The dog, for example, is frequently born with the disease, or contracts it immediately after birth, and according to the opinion of many naturalists, all the smaller breeds of the canine species, as terriers, poodles, &c., owe their dwarfish size to an hereditary rachitis.

M. Heusinger next proceeds to describe glanders and farcy; but as the author has nothing new to offer on the subject, and as we have discussed these diseases at length in a recent number of this Journal (British and Foreign Medical Review, XLIII), we shall pass on to the cachectic maladies which follow. Hemorrhoids have been described by many authors as a common disease of animals, but the author thinks that hemorrhagic discharges by the rectum from other sources (tumours, &c.) have been mistaken for this, as he himself has only observed it in the carnivori. Toggia, an Italian writer, however, describes it as a frequent disease amongst oxen.

The early writers describe arthritis as a very common affection of our domestic animals; but in a great many instances they evidently confound rickety affections with articular diseases, especially in the cases of lambs and calves. The dog, however, is really attacked by the "gouty disease" (which term is evidently, however improperly, meant to include articular rheumatism). Rheumatic diseases are extremely frequent amongst all the domestic animals. The most common form of rheumatism in animals, and particularly in horses, is the disease called "founder," *la fourbure* of the French writers. Veterinarians recognise two varieties, the rheumatic and the inflammatory; but the former is merely simple rheumatism, and the latter an inflammatory form of that disease. Rheumatism of the joints and vertebræ is often the cause of exostosis and anchylosis in horses and oxen. Dogs are particularly subject to rheumatism of the spinal column, which generally terminates in permanent paralysis of the posterior extremities. According to M. Huzard, scurvy is a common affection in dogs and other of the carnivori, when these animals are fed almost exclusively on meat and are not exercised. The author has not observed this disease, and is doubtful of its existence.

Cachectic maladies of a septic tendency are, according to M. Heusinger, much more frequent amongst herbivorous animals than amongst the carnivori and man. The "*fièvre charbonneuse*" of animals corresponds in some sort to the typhoid fevers in the human species, and although the former are not subject to the depressing passions and nervous shock or weakness which so often causes the disease in man, they are nevertheless incapable of resisting many atmospheric influences, as negative electricity, excessive heat or cold, marsh miasma, &c.

All our domestic animals are subject to contagious discharges, vegetations, and ulcerations of the genital organs, which are transmissible by coition, and exhibit a resemblance to syphilitic affections. In the author's opinion these diseases are nearly in the same condition as the syphilitic affections of man were before the close of the fifteenth century. M. Heusinger discards the opinion of several writers, that the disease has been originally propagated from the human species to the brute. Carcinomatous diseases are not so common in animals as in man.

NERVOUS SYSTEM. *Diseases of the brain.* Serous apoplexy and chronic hydrocephalus have been observed in animals, but the latter much more frequently than the former. Pseudo-morphosis of the brain is much more common than the preceding, especially the transformation of a new product, which consists of a kind of cellular and cartilaginous tissue, into a state of ossification. This condition has been erroneously described by authors under the name of enchondroma. Hydatids are frequently met with. The *cysticercus* is found oftener in the brain of the hog than in that of any other of the domestic animals. The *cœnurus cerebralis* is very common in the sheep, less so in the goat, and rarely found in horses and oxen. Congestion and hemorrhage of the brain occur frequently in animals. M. Heusinger has even observed the latter affection in birds. Hemorrhage of the brain, however, is not so common as it is in man, which circumstance is no doubt mainly owing to the more natural life which animals lead. Nervous apoplexy also occurs in the brute, but much more rarely than in man.

*Diseases of the spinal marrow.* These complaints are extremely frequent amongst our domestic animals. Inflammation of the membranes is very common. Dropsy of the spinal canal is more frequent amongst the domestic animals than in man, and is often a congenital disease in lambs. Since the beginning of the present century a kind of plague has been frequently observed amongst sheep, known under the name of the trembling disease (*maladie tremblante*), which is the result of the pathological condition just described. Effusion of matter is a frequent occurrence in horses, and is often fatal. The author has observed the case of a mare who died suddenly from the effusion of pus from a lumbar abscess into the spinal canal. Atrophy of the spinal marrow occurs frequently in horses, dogs, and sheep. Inflammatory softening of its substance is very common in all our domestic animals.

M. Dupuy thinks he has observed the roaring disease, "*cornage,*" in horses, in which the recurrent nerves were compressed by indurated lymphatic ganglions. Mr. Ferguson, of Dublin, has observed the same condition. This disease resembles *laryngismus stridulus* in man. Paralysis is very common in animals. There is a singular epizootic disease of cattle of this kind, called by the Germans *sterzwurm*, which appears to be unknown to veterinarians of the present day. It generally terminates in gangrene. Trismus and tetanus are singularly common affections in horses, but rarely occur in the other domestic animals. The nature of these diseases is as obscure in the brute as in the human subject. Convulsions and epilepsy occur frequently in dogs, cats, and birds. Chorea has been observed in dogs. Canine madness (*rabies canina*) originates in the dog species, and also, according to some writers, in the cat. It has been observed as an original disease in the dog, the wolf, the fox, and jackal. It is sporadic and epizootic, and may be transmitted to all quadrupeds, to birds, and man, but its transmissibility from animals in which it was not an original disease is, according to M. Heusinger, very problematical.

Mental diseases occur in animals as well as in man, according to the author, who is supported in this view by M. Tscheulin, a German writer on the nervous diseases of animals. M. Heusinger believes that "instinct" depends for its healthy existence upon the organization of the nervous system, and, as that organization may be disturbed or deranged, it follows as a corollary that derangement of the instinct, or mental disease, will follow. The author considers that some light might be thrown on the *morbi mentis* in man by the study and observation of these melancholy disorders in the brute. The *furor uterinus* and the mania for destroying their young are subjects worthy of the special attention of the comparative pathologist.

*Diseases of the eye* in animals have been so ill-described by veterinarians, that the author complains he cannot even give a complete compilation of them. The same may be said of diseases of the ear, which is the last subject described in the 'Comparative Nosography.'

We have now completed our analysis of M. Heusinger's memoir. The original, being in a tabular form, loses much of its force in being transposed into consecutive sentences, and our abstract is of necessity abrupt in many places; but still the substance and meaning of the author are preserved throughout, and the reader is furnished with a number of facts, as interesting and important as they are unique.

